# Long-Term Outcome After Resection of Hepatic and Pulmonary Metastases in Multivisceral Colorectal Cancer

**DOI:** 10.3390/cancers16223741

**Published:** 2024-11-05

**Authors:** Christopher Berlin, Geoffroy Andrieux, Magdalena Menzel, Gabriel J. Stöger, Andreas Gengenbach, Luisa Schäfer, Hans C. Hillebrecht, Rebecca Kesselring, Uyen-Thao Le, Stefan Fichtner-Feigl, Philipp A. Holzner

**Affiliations:** 1 Department of General and Visceral Surgery, Medical Center–University of Freiburg, Faculty of Medicine, University of Freiburg, 79106 Freiburg im Breisgau, Germany; magdalena.menzel@uniklinik-freiburg.de (M.M.); gabriel.stoeger@uniklinik-freiburg.de (G.J.S.); andreas.gengenbach@uniklinik-freiburg.de (A.G.); luisa.schaefer@uniklinik-freiburg.de (L.S.); christian.hillebrecht@uniklinik-freiburg.de (H.C.H.); rebecca.kesselring@uniklinik-freiburg.de (R.K.); stefan.fichtner@uniklinik-freiburg.de (S.F.-F.); 2 German Cancer Consortium (DKTK) Partner Site, 79106 Freiburg im Breisgau, Germany; 3 IMMediate Advanced Clinician Scientist-Program, University of Freiburg, 79106 Freiburg im Breisgau, Germany; 4 Institute of Medical Bioinformatics and Systems Medicine, Medical Center–University of Freiburg, 79106 Freiburg im Breisgau, Germany; geoffroy.andrieux@uniklinik-freiburg.de; 5 Department of Thoracic Surgery, Medical Center–University of Freiburg, Faculty of Medicine, University of Freiburg, 79106 Freiburg im Breisgau, Germany

**Keywords:** colorectal cancer metastasis, metastasis surgery, liver and lung metastasis, multivisceral colorectal cancer

## Abstract

Background/Objectives: Colorectal cancer (CRC) with hepatic (CRLM) and pulmonary metastases (CRLU) presents a significant clinical challenge, leading to poor prognosis. Surgical resection of these metastases remains controversial because of limited evidence supporting its long-term benefits. To evaluate the impact of surgical resection of both hepatic and pulmonary metastases on long-term survival in patients with multivisceral metastatic colorectal cancer, this retrospective cohort study included 192 patients with UICC stage IV CRC treated at a high-volume academic center. Methods: Patients were divided into two groups: those who underwent surgical resection of both hepatic and pulmonary metastases (n = 100) and those who received non-surgical treatment (n = 92). Propensity score matching was used to adjust for baseline differences. The primary outcome was overall survival (OS). Results: Unadjusted analysis showed a significant OS benefit in the surgical group (median OS: 6.97 years) compared with the conservative group (median OS: 2.17 years). After propensity score matching, this survival advantage persisted (median OS: 5.58 years vs. 2.35 years; HR: 0.3, 95% CI: 0.18–0.47, *p* < 0.0001). Conclusions: Surgical resection of hepatic and pulmonary metastases in multivisceral metastatic CRC significantly improves long-term survival, supporting an aggressive surgical approach in selected patients.

## Simple Summary

To assess the potential therapeutic efficacy of surgical intervention for hepatic and pulmonary metastases in colorectal cancer, we conducted a retrospective analysis of long-term outcomes in 192 patients treated at a high-volume academic medical center in Germany. The cohort comprised 100 patients who underwent resection of metastatic lesions in the liver and lungs and 92 patients who received non-surgical management for metastatic disease. All patients underwent primary tumor resection, and the study population included cases of both synchronous and metachronous disease progression. Propensity score-matched survival analysis demonstrated significant long-term survival advantages following hepatic and pulmonary metastasectomy in patients with multivisceral colorectal cancer.

## 1. Introduction

Colorectal cancer (CRC) stands as the third most prevalent cancer worldwide, contributing significantly to cancer-related mortality. Initial diagnostics reveal distant organ metastases in 25–30% of patients, predominantly affecting the liver, peritoneum, and lungs [1,2,3]. Despite efforts in prevention and early detection, widespread metastatic disease remains challenging, necessitating decisions based on scarce evidence and individualized considerations in multidisciplinary tumor boards. Outcomes for metastatic CRC treated with chemotherapy alone remain poor. A systematic review of modern chemotherapy regimens in metastatic CRC reported median overall survival ranging from 13.8 to 19.5 months. Three-year survival rates with chemotherapy alone are typically less than 20%, and 5-year overall survival (OS) rates are approximately 5% [4].

For CRC patients with liver-only metastases (CRLMs), recent series report a favorable 5-year OS after hepatic metastasectomy, reaching up to 78% [5,6,7,8,9,10]. Liver resection, even after neoadjuvant chemotherapy or employing a liver-first approach, is a cornerstone in the multimodal treatment of metastatic CRC (mCRC). Advancements in hepatic resection techniques, notably portal vein embolization and associating liver partition with portal vein ligation for staged hepatectomy, have expanded the feasibility of invasive procedures and iterative liver resections in the context of disease recurrence and progression [11,12,13]. However, therapeutic approaches for patients with extrahepatic tumor spread, such as pulmonary or peritoneal metastases, remain more individualized because of conflicting data and smaller cohort sizes.

Pulmonary metastases (CRLU), whether synchronous or metachronous, present challenges, with some patients receiving palliative treatment despite feasible tumor clearance. Selected patients with pulmonary metastases, either with or without hepatic involvement, have shown promising survival outcomes with repeated surgical interventions in conjunction with modern chemotherapy [14,15,16,17]. As in hepatobiliary surgery, morbidity and mortality of pulmonary resection have decreased with the growing experience of surgeons in high-volume centers [15,18,19,20,21].

Despite the established efficacy of isolated hepatic resection for CRLM, the optimal management strategy for patients presenting with synchronous or metachronous pulmonary metastases, or concomitant extrahepatic disease, remains undefined. In particular, there is a paucity of clinical data and standardized treatment algorithms for the most prevalent combination of metastatic sites: the liver and lungs. The management of patients with both hepatic and pulmonary metastases presents a unique challenge, as it represents a more advanced stage of the disease. However, emerging evidence suggests that carefully selected patients may benefit from aggressive surgical management [22]. A systematic review by Salah et al. reported 5-year overall survival rates of 19–56% for patients who underwent pulmonary metastasectomy following previous liver resection for metastases [23]. These encouraging results have prompted further investigation into prognostic factors and patient selection criteria to optimize outcomes in this complex patient population [24].

Analysis of the Surveillance, Epidemiology, and End Results (SEER) Program register data suggests inconclusive evidence regarding substantial improvements in overall survival following surgical resection of both CRLM and CRLU across specialized and non-specialized centers in the United States [3]. This dataset encompasses a heterogeneous mix of university medical centers and community hospitals, where clinical decision-making practices vary considerably, potentially leading to disparate outcomes. Additionally, only 262 out of 10,325 patients underwent resection of hepatic and pulmonary metastases, and it remains unclear whether relapse metastasectomies were included or not. The authors concluded that resection of hepatic and pulmonary metastases does not improve cancer-specific survival. Notably, the cohort comprises 30.9% and 18.9% of patients without chemotherapy in the resection group and the control group, respectively. Furthermore, around 25% of patients are non-Caucasian.

Consequently, the primary objective of our study was to evaluate the potential long-term (>10 years) survival benefits of interdisciplinary stratification for surgical metastasis resection in a highly specialized academic center with expertise in both hepatobiliary and thoracic surgery. In this setting, clinical decision-making is informed by surgical proficiency for both anatomical locations, potentially offering insights into optimized management strategies for the iterative surgical treatment of this complex patient population. Hence, our study is characterized by an extensive observational timeframe exceeding 10 years. This prolonged period of observation is necessitated by two key factors: first, the treatment history for individual patients frequently spans more than five years, reflecting the complex and often protracted nature of managing metastatic colorectal cancer. Second, surveillance protocols are reinitiated following each treatment of metastatic relapse, resulting in cumulative follow-up periods that often extend beyond a decade. This extended timeframe enables a comprehensive assessment of long-term outcomes, including the efficacy of iterative interventions, patterns of disease recurrence, and the durability of treatment responses, thereby providing critical insights into the long-term viability and impact of our management approach.

## 2. Materials and Methods

### 2.1. Patient Cohort

All 192 patients in the analyzed cohort were treated at the Freiburg University Medical Center between 1990 and 2019. Clinical data of both treatment groups were collected at the Department of General and Visceral Surgery and the Comprehensive Cancer Center Freiburg (CCCF), including survival data.

### 2.2. Study Design

Patients aged over 18 years and diagnosed with colorectal cancer were eligible for analysis. All patients underwent surgical resection of the primary tumor (not necessarily at the Freiburg University Medical Center). All T-stages of the primary tumor were included, except in situ carcinomas. All patients developed synchronous or metachronous hepatic and pulmonary metastasis during follow-up.

### 2.3. Statistical Methods

We summarized and compared baseline characteristics between the control (Ctrl) and resection groups using the tableone (v0.13.2) R package. One numeric feature (age) was compared between groups using the Wilcoxon rank-sum test. Eight nominal features were compared using Fisher’s exact test. For each feature, a *p*-value of less than 0.05 was considered indicative of significant differences between the groups.

Propensity matching was conducted using the matchit R package (v4.5.5). The nearest neighbor matching method was employed, with propensity score differences serving as the distance metric, estimated through logistic regression. Covariates included in the propensity score estimation comprised age at diagnosis, gender, presence of synchronous and metachronous hepatic and pulmonary metastasis, and the sequence of metastasis according to anatomical locations. To enhance matching quality and mitigate potential bias, a caliper was applied, ensuring that matched units did not exceed a specified threshold of propensity score difference. The caliper was set at 0.05 times the standard deviation of the propensity score. Out of the original 192 samples, 70 were successfully matched.

Survival outcomes were assessed using Kaplan–Meier survival curves and the Cox proportional hazards (Coxph) model. The Kaplan–Meier method was employed to estimate survival probabilities over time, and differences between groups were evaluated using the log-rank test. For multivariate analysis, we fitted a Coxph model to evaluate the effect of surgical resection, age, gender, Pn status, V status, KRAS mutational status, and systemic chemotherapy as covariates on survival. Hazard ratios (HRs) and 95% confidence intervals (CIs) were reported for each covariate. All survival analyses were performed using the survival (v3.7.0) and survminer (v0.4.9) R packages.

### 2.4. Study Approval

This human retrospective study was conducted according to local regulations at the University of Freiburg Medical Center and the Declaration of Helsinki.

## 3. Results

### 3.1. Patients Characteristics

A total of 192 patients diagnosed with mCRC were included in the analysis. All patients were treated at the CCCF during the course of their disease and underwent surgical resection of the primary tumor at Freiburg University Medical Center or ex domo. Only patients with synchronous or metachronous development of CRLM and CRLU were included in the analysis. Hepatic and pulmonary metastasectomy with or without systemic therapy (yes: 65%, no: 20%, unknown: 15%) during the course of the disease was performed in 100 patients. In total, 92 patients underwent systemic therapy only. All therapeutic decisions were based on interdisciplinary expertise in our high-volume academic medical center. Overall, 108 patients suffered from synchronous (<9 months from time of diagnosis) and 84 patients from metachronous (>9 months from time of diagnosis) hepatic mCRC. The classification criteria for synchronous and metachronous diseases were established in accordance with the institutional CCCF guidelines. The median overall survival time for the complete cohort was 5.58 (SD: 5.06) years. All patient characteristics are depicted in Table 1.

### 3.2. Surgical Resection of Hepatic and Pulmonary Metastases Impacts Overall Survival

**Figure 1:**
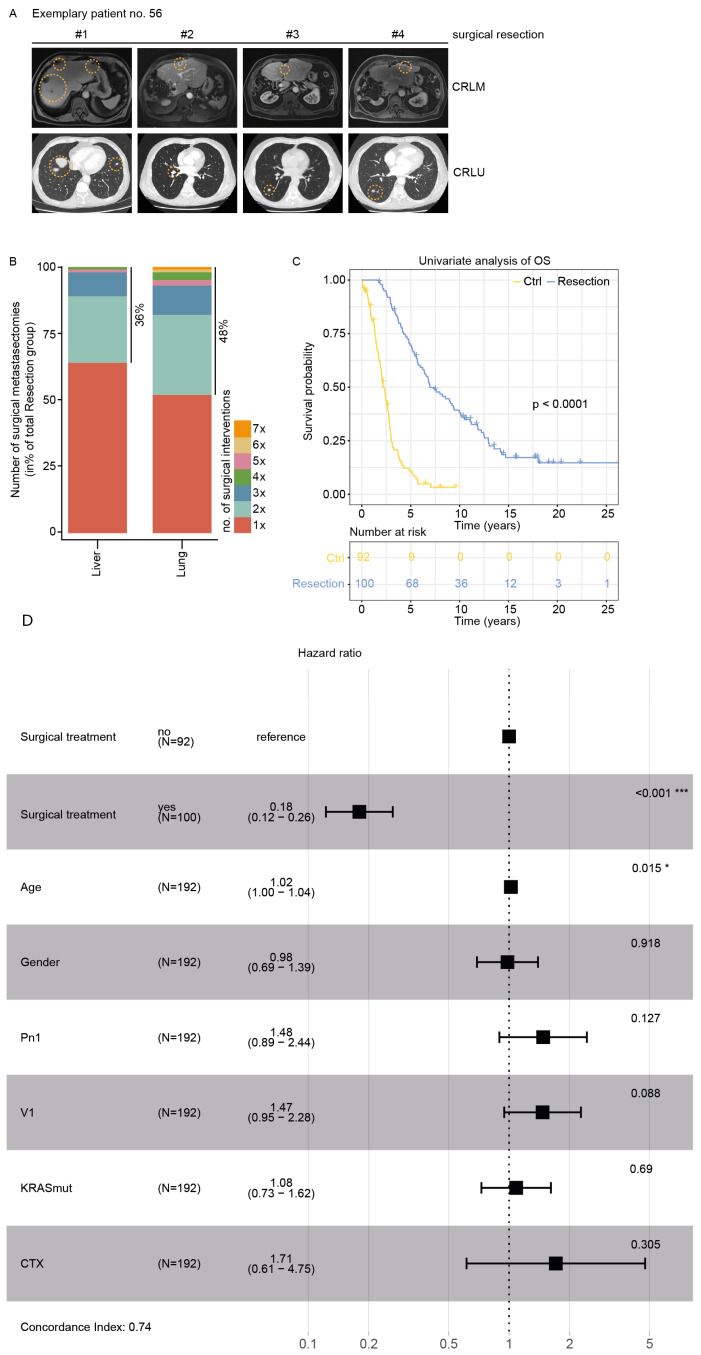
(**A**) Exemplary CT and MRI images of patient no. 56 who underwent four surgical resections of CRLMs and four surgical resections of CRLUs. Exemplary CRLMs and CRLUs (orange dashed circle), as well as corresponding relapse tumors (orange dashed circle), are shown. (**B**) Barplot for the number of surgical metastasectomies per site in % of the resection group. The number of surgical metastasectomies is color-coded. (**C**) Kaplan–Meier curve of univariate overall survival in the Ctrl and treatment group. n = 192. *p* < 0.0001. “OS”: overall survival. (**D**) Forest plot of HRs from the multivariate Cox proportional hazards model. The plot shows the estimated HRs and 95% CIs for each covariate included in the model. The horizontal line represents the null value (HR = 1), where no effect is observed. “*” *p* value ≤ 0.05, “***” *p* value ≤ 0.001.

In this retrospective cohort study, we evaluated the efficacy of surgical intervention versus conservative management in patients with hepatic and pulmonary metastases. Our analysis encompassed 192 subjects, where 100 patients underwent surgical resection, and 92 received conservative treatment. Among the surgical cohort, a substantial proportion underwent multiple interventions: 36% had more than one hepatic resection, while 48% underwent repeated pulmonary metastasectomies (Figure 1A,B). This high rate of reintervention underscores the aggressive surgical approach employed in our institution for oligometastatic disease. To assess the impact of surgical intervention on patient outcomes, we conducted an unadjusted univariate analysis of overall survival (OS). The results revealed a statistically significant survival benefit associated with surgical management. Patients in the conservative treatment arm exhibited a median OS of 2.17 years, whereas those in the surgical resection group achieved a median OS of 6.97 years (Figure 1C). The multivariate Cox proportional hazard ratio (HR) for mortality in the surgical group compared with the conservative group was 0.18 (95% confidence interval (CI): 0.12–0.26), indicating a substantial reduction in mortality risk associated with surgical intervention. Furthermore, the analysis revealed surgical treatment as the most prognostically relevant covariate, underscoring its pivotal role in patient outcomes. Age also emerged as a significant factor, albeit with a lower level of significance and a modest increase in the hazard ratio (Figure 1D). These findings suggest that aggressive surgical management, including repeated resections when indicated, may confer a significant survival advantage in selected patients with hepatic and pulmonary metastases.

### 3.3. Statistical Analysis of Baseline Characteristics

To address potential confounding factors arising from statistically significant imbalances in baseline patient characteristics between the treatment groups, we performed statistical tests to compare the variables across the strata groups (Ctrl and resection). Our analysis revealed significant differences between the surgical and conservative treatment cohorts with respect to several disease progression patterns, clinicopathologic characteristics, and systemic interventions. Specifically, we observed disparities in the prevalence of metachronous versus synchronous metastatic disease, as well as variations in the location-dependent metastatic sequence, Pn and V status, applications of systemic chemotherapy (CTX), and KRAS mutational status (Table 2). These findings underscore the importance of accounting for such factors when interpreting the observed survival differences.

### 3.4. Propensity Score Matching

**Figure 2:**
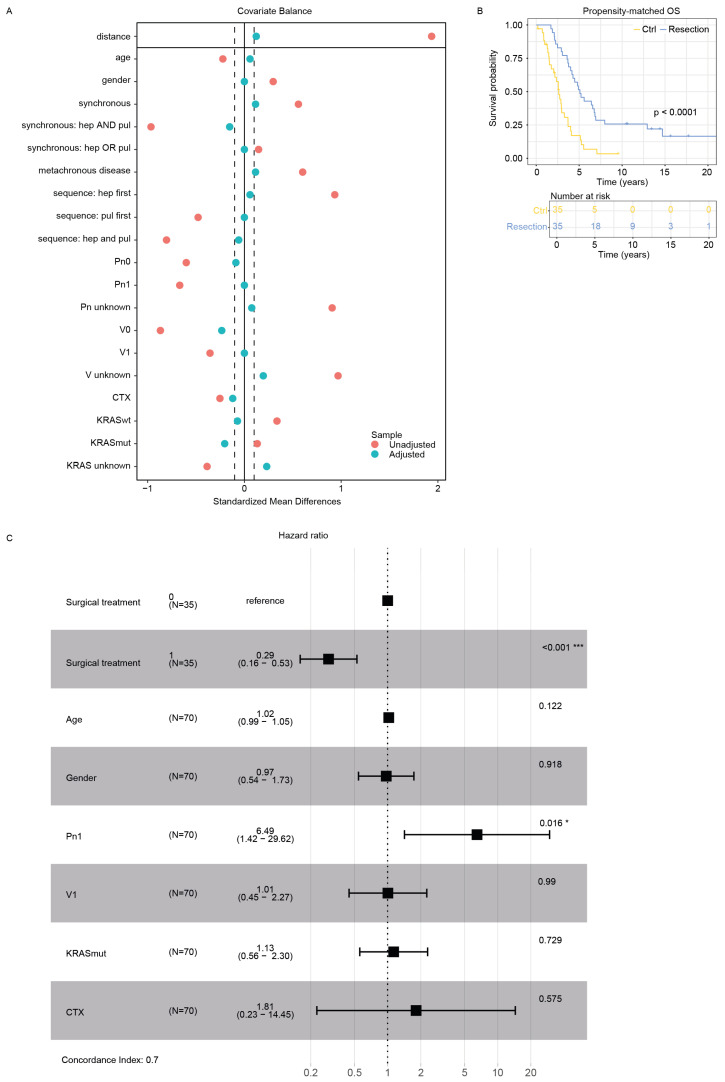
(**A**) Balance plot for propensity score matching for respective parameters. (**B**) Kaplan–Meier curve of propensity score-matched overall survival in the Ctrl and treatment group. n = 70. *p* < 0.0001. (**C**) Forest plot of HRs from the multivariate Cox proportional hazards model. The plot shows the estimated HRs and 95% CIs for each covariate included in the model. The horizontal line represents the null value (HR = 1), where no effect is observed. “*” *p* value ≤ 0.05, “***” *p* value ≤ 0.001.

Given the statistically significant impact on prognosis revealed by multivariable logistic regression analysis in our cohort of hepatopulmonary mCRC patients (Figure 1D, Table 2), we implemented propensity score matching to adjust for observed covariates. This approach allowed us to balance potential confounding variables between treatment groups, including age at diagnosis, gender, synchronous versus metachronous presentation, and location-dependent metastatic sequence, as well as clinicopathologic characteristics, systemic CTX, and KRAS mutational status (Figure 2A). The propensity score matching procedure resulted in a refined cohort of 70 patients, with 35 patients in each treatment arm. This balanced sample size ensured adequate statistical power while mitigating selection bias. Subsequent analysis of OS in the matched cohorts corroborated our initial findings, further substantiating the protective effect of surgical metastasectomy. The adjusted survival analysis revealed a median OS of 5.09 years in the surgical resection group compared with 2.62 years in the conservative treatment group (Figure 2B). The multivariate Cox proportional HR of 0.29 (95% CI: 0.16–0.53) indicated a 70% reduction in the risk of mortality for patients undergoing surgical resection. Thus, we reanalyzed the propensity-matched cohort using a multivariate Cox proportional hazard ratio model. As depicted already in the unadjusted cohort (Figure 1D), surgical resection remained the most prominent protective covariate with the highest significance level. Furthermore, a positive Pn1 status was significantly associated with an increased HR (Figure 2C). This persistent and significant survival advantage associated with surgical intervention, even after controlling for key prognostic factors, reinforces the potential benefits of aggressive surgical management in carefully selected patients with hepatic and pulmonary CRC metastases. The confidence interval in this adjusted analysis suggests conserved precision in our estimate of treatment effect, enhancing the statistical robustness of our conclusions. These findings align with the growing body of evidence supporting an aggressive approach to oligometastatic disease in colorectal cancer, challenging traditional paradigms of systemic therapy alone for metastatic disease.

## 4. Discussion

Our analysis reveals that surgical resection of both hepatic and pulmonary metastases is associated with a marked improvement in long-term OS compared with conservative treatment. This is consistent with previous findings that indicate substantial survival benefits from hepatic metastasectomy [5,7,25]. Notably, our study underscores that this benefit extends to patients with pulmonary metastases.

The use of propensity score matching to address selection bias reinforces these findings. After adjusting for confounders such as age, gender, the sequence and location of metastases, and systemic therapy, as well as clinicopathological features and KRAS mutational status, surgically treated patients continued to exhibit significantly improved long-term survival compared with those receiving conservative treatment only. The emergence of pN1 as a significant prognostic factor in the propensity-matched analysis likely reflects the enhanced detection of subtle prognostic indicators in a more balanced cohort. This finding aligns with the established literature on pN1’s importance in CRC stratification, while the persistent significance of surgical treatment across both analyses underscores its robust impact on patient outcomes. Thus, our results align with the recent literature that supports the effectiveness of surgical approaches in managing complex metastatic CRC cases [26,27].

Our findings are in concordance with several recent studies highlighting the survival benefits of surgical interventions for metastatic CRC [28,29]. Recently, a nation-wide register-based analysis by Engstrand et al. [26] reported protective surgical treatment of synchronous CRLM and CRLU. This is consistent with our long-term (>10 years) observation that metastatic resection, both hepatic and pulmonary, significantly extends OS in our high-volume medical center.

Still, our study’s focus on combined hepatic and pulmonary metastases and its impact on long-term survival is less explored in the literature. While most studies also highlight improved outcomes for pulmonary metastasectomy [30,31,32], they often focus on isolated metastases rather than combined hepatic and pulmonary involvement. Few studies focused on the special occasion of multilocular metastasectomy in the liver and lungs, and some also failed to show improvement in OS upon this specific surgical treatment in comparison to conservative treatment [3,33,34,35,36]. While the SEER analysis [3] initially encompasses a larger patient cohort than our study, its multi-institutional nature and lack of information regarding decision-making expertise at each participating center present limitations. After adjusting for potential confounders like site of the primary tumor, metastatic site, T-stage, nodal status, grading, chemotherapy, year of diagnosis, age, gender, ethnicity, and marital status, Siebenhüner et al. reported no statistically significant difference in median survival between patients undergoing resection of both hepatic and pulmonary metastases (1.5 years) and those receiving conservative treatment (1.2 years). In contrast, our study demonstrates a median OS of 5.09 years for patients undergoing sometimes iterative resection at both sites, with more extensive adjustments for confounders (synchronous/metachronous disease, sequence of progression, Pn status, V status, systemic treatment, and KRAS mutational status). Furthermore, the SEER database lacks information on treatment for recurrence, which introduces variability based on the expertise of each contributing center. Unlike the SEER analysis, where the time point for survival analysis remains ambiguous, our study clearly defines initial diagnosis as the threshold for overall survival assessment.

Of particular significance, and to our knowledge, there is a notable absence of publicly available data on long-term survival (exceeding 10 years) for patients who have undergone repeated surgical interventions at both hepatic and pulmonary metastatic sites. This is especially relevant given that the treatment course for these patients often spans more than five years. Our study addresses this critical gap in the literature by providing comprehensive long-term follow-up data on this unique patient cohort. The extended timeframe of our analysis allows for a more nuanced understanding of the cumulative impact of iterative surgical interventions on patient outcomes. By capturing survival data beyond the typical 5-year benchmark, we offer insights into the durability of treatment responses and the potential for sustained disease control in patients with metastatic colorectal cancer involving both the liver and lungs. Our findings contribute valuable insights into the benefits of iterative resection in the context of both types of metastases, reflecting a trend towards integrated treatment strategies.

Despite these promising results, several limitations warrant consideration. Although propensity score matching was employed to mitigate bias, inherent selection biases still exist. The decision to proceed with surgical resection was likely influenced by factors not fully captured in our dataset, such as patient performance status and the exact biological behavior of the metastases like, e.g., genetic and epigenetic states and modulation of the immunological tumor microenvironment [37,38,39] as well as local surgical long-term experience. The retrospective design of this study inherently limits the ability to establish causality.

While we observed significant differences in survival, prospective randomized trials are needed to validate these findings and refine treatment protocols. Furthermore, the heterogeneity in metastatic patterns and treatment responses highlights the need for personalized treatment approaches. Future research should focus on identifying specific subgroups of patients who may benefit most from surgical interventions, incorporating molecular and genetic profiles to tailor treatments more precisely.

## 5. Conclusions

In conclusion, our study supports the role of surgical resection in improving overall survival for CRC patients with synchronous or metachronous hepatic and pulmonary metastases. The evidence presented here reinforces the importance of an interdisciplinary and highly specialized approach and highlights the need for ongoing research to optimize treatment strategies. As the field progresses, integrating novel therapies and refining surgical techniques will be crucial in further enhancing outcomes for patients with metastatic CRC.

## Figures and Tables

**Table 1:** Patient characteristics of the complete cohort. “hep”: hepatic; “pul”: pulmonary; “undiff.”: undifferentiated; “wt”: wildtype; “mut”: mutated; “OS”: overall survival; “SD”: standard deviation.

Patients Included	192				
Metastasectomy (%)	no	yes			
	92 (47.9)	100 (52.1)			
Age at primary tumor diagnosis in years (SD)	60.35 (11.34)				
Gender (%)	Female	Male			
	128 (66.7)	64 (33.3)			
Metastasis sequence (%)	synchronous	metachronous			
	108 (56.2)	84 (43.8)			
Synchronous hepatic or pulmonary metastasis	hep AND pul	hep OR pul	metachronous		
	66 (34.4)	42 (21.8)	84 (43.8)		
Metastasis sequence and location (%)	hep first	pul first	hep and pul		
	71 (37.0)	10 (5.2)	111 (57.8)		
Pathology					
Primary tumor grading (%)	1	2	3	undiff.	
	2 (1.0)	157 (81.8)	32 (16.7)	1 (0.5)	
pT (%)	1	2	3	4	unknown
	5 (2.7)	15 (7.8)	120 (62.5)	36 (18.7)	16 (8.3)
pN (%)	0	1	2		unknown
	57 (29.7)	65 (33.9)	55 (28.6)		15 (7.8)
		0	unknown		
L1 (%)	47 (24.5)	32 (16.6)	113 (58.9)		
V1 (%)	29 (15.2)	60 (31.2)	103 (53.6)		
Pn1 (%)	23 (12.0)	41 (21.4)	128 (66.6)		
KRAS (%)	wt	mut	unknown		
	26 (13.5)	39 (20.3)	127 (66.2)		
Systemic therapy (%)	yes	no			
	186 (96.9)	6 (3.1)			
Primary tumor location (%)	Right	Left and rectum	bilocular		
	32 (16.7)	154 (80.2)	6 (3.1)		
Primary tumor (%)	Colon	Rectum	bilocular		
	100 (52.1)	86 (44.8)	6 (3.1)		
OS in years (SD)	5.58 (5.06)				

**Table 2:** Statistical test between treatment groups comparing age, gender, and disease progression parameters. “SMD”: standardized mean difference.

Baseline	Ctrl	Resection	*p*	Test SMD
n	92	100		
Age at diagnosis (SD)	61.46 (12.90)	59.32 (9.63)	0.191	0.188
Gender: male (%)	55 (59.8)	73 (73.0)	0.074	0.283
Metachronous disease (%)	25 (27.1)	57 (57.0)	<0.001	0.581
Synchronous disease (%)			<0.001	0.843
hep and pul	49 (53.3)	17 (17.0)		
hep or pul	18 (19.6)	26 (26.0)		
Sequence (%)			<0.001	1.103
hep first	12 (13.0)	59 (59.0)		
pul first	8 (8.7)	2 (2.0)		
hep and pul	72 (78.3)	39 (39.0)		
Pn (%)			<0.001	0.777
Pn0	29 (31.5)	12 (12.0)		
Pn1	18 (19.6)	10 (10.0)		
Unknown	45 (48.9)	83 (83.0)		
V (%)			<0.001	0.948
V0	44 (47.8)	16 (16.0)		
V1	19 (20.7)	10 (10.0)		
Unknown	29 (31.5)	74 (74.0)		
CTX (%)			0.049	0.357
Yes	92 (100.0)	94 (94.0)		
No	0 (0.0)	6 (6.0%)		
KRAS (%)			0.007	0.468
KRASwt	6 (6.5)	20 (20.0)		
KRASmut	16 (17.4)	23 (23.0)		
Unknown	70 (76.1)	57 (57.0)		

## Data Availability

All raw data and code are available from the authors upon reasonable request.
